# Supplementary material for: Characterization of an IDH1 R132H Rabbit Monoclonal Antibody, MRQ-67, and Its Applications in the Identification of Diffuse Gliomas
Source: Antibodies (Basel). 2023 Feb 6;12(1):14. doi: 10.3390/antib12010014 (PMC9944093; doi:10.3390/antib12010014)
Supplement: Supplementary file 1 [file antibodies-12-00014-s001.zip › antibodies-2159119-supplementary.pdf]

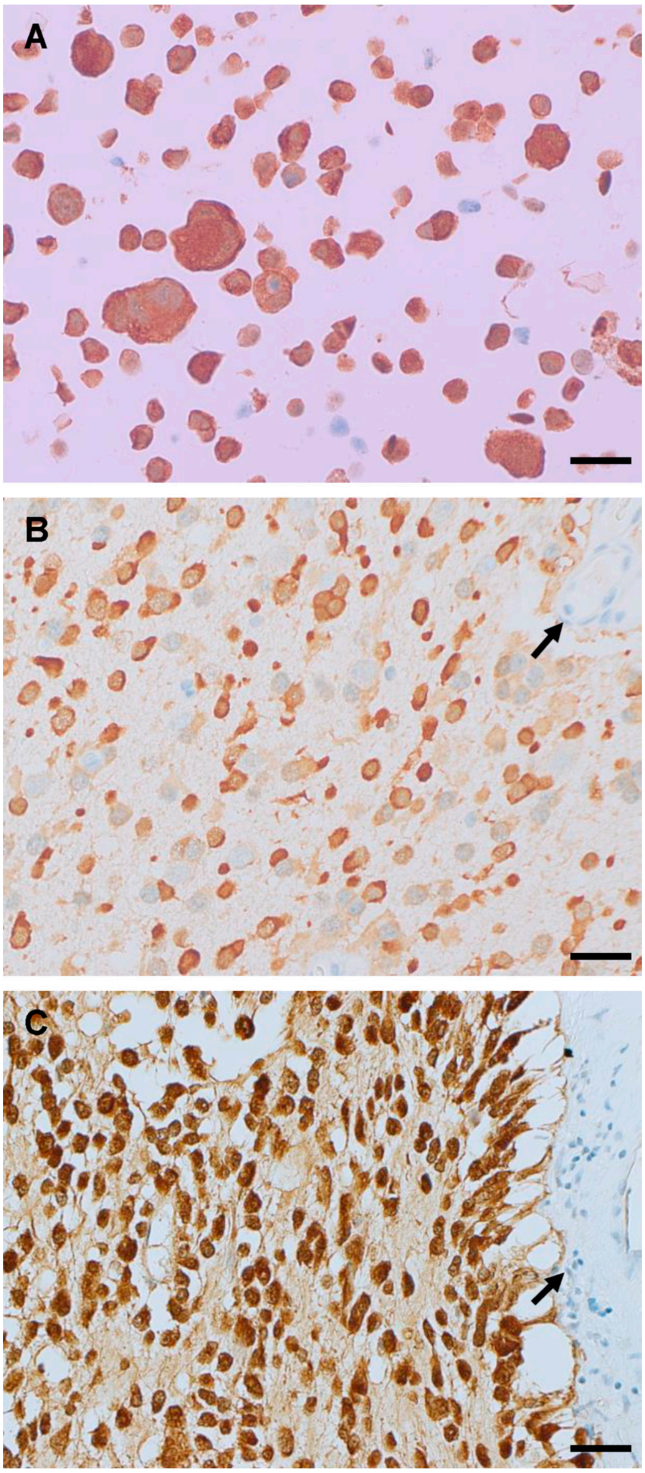

Figure S1. IDH1 R132H staining pattern as demonstrated by IHC with MRQ-67. (**A**) Glioma cells in the BT142 mut/- cell pellet block stained positive, with the immunoreactivity in cytoplasmic and nuclear spaces. (**B**,**C**) Tissue samples from an oligodendroglioma (**B**) and a secondary glioblastoma (**C**) showing a cytoplasmic and nuclear stain in tumor cells, but not in blood vessels (arrow). UltraView, visualized with DAB, and counterstained with hematoxylin. Scale bar = 50 μm.

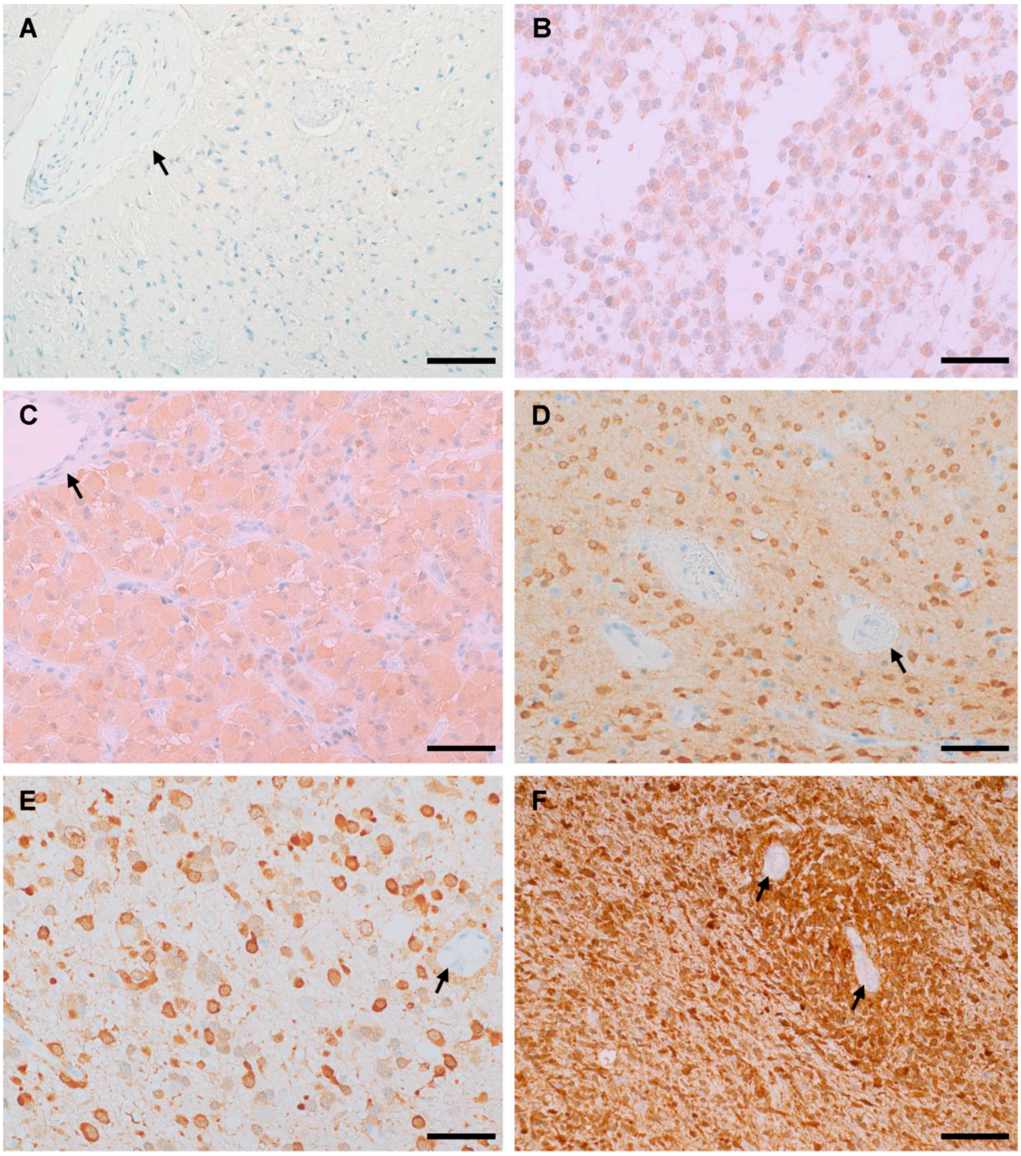

Figure S2. Result assessment of the immunostaining on diffuse gliomas as shown with MRQ-67. Results were graded as negative (0; **A**) and positive (**B**-**F**), and immunoreactivity levels were scored as 1 (**B**), 2 (**C**), 3 (**D**), 3.5 (**E**) and 4 (**F**). The reactivity is localized in tumor cells, but not in blood vessels (arrow). UltraView, visualized with DAB, and counterstained with hematoxylin. Scale bar = 100 μm.
